# Zirconia Reduced Graphene Oxide Nano-Hybrid Structure Fabricated by the Hydrothermal Reaction Method

**DOI:** 10.3390/ma13030687

**Published:** 2020-02-04

**Authors:** Anton Smirnov, Nestor Washington Solís Pinargote, Nikita Peretyagin, Yuri Pristinskiy, Pavel Peretyagin, José F. Bartolomé

**Affiliations:** 1 Spark Plasma Sintering Research Laboratory, Moscow State University of Technology “STANKIN”, Vadkovsky per. 1, Moscow 127055, Russia; nw.solis@stankin.ru (N.W.S.P.); n.peretyagin@stankin.ru (N.P.); y.pristinskiy@stankin.ru (Y.P.); p.peretyagin@stankin.ru (P.P.); 2 Instituto de Ciencia de Materiales de Madrid (ICMM), Consejo Superior de Investigaciones Científicas (CSIC), C/ Sor Juana Inés de la Cruz 3, 28049 Madrid, Spain

**Keywords:** one-step hydrothermal synthesis, graphene oxide, zirconia nanoparticles, zirconia-reduced graphene oxide nanocomposite

## Abstract

In this work, we report an available technique for the effective reduction of graphene oxide (GO) and the fabrication of nanostructured zirconia reduced graphene oxide powder via a hydrothermal method. Characterization of the obtained nano-hybrid structure materials was carried out using a scanning electron microscopy (SEM), transmission electron microscopy (TEM), X-ray diffraction (XRD), Raman spectroscopy, X-ray photoelectron spectroscopy (XPS) and Fourier-transform infrared spectroscopy (FTIR). The confirmation that GO was reduced and the uniform distribution of zirconia nanoparticles on graphene oxide sheets during synthesis was obtained due to these techniques. This has presented new opportunities and prospects to use this uncomplicated and inexpensive technique for the development of zirconia/graphene nanocomposite powders.

## 1. Introduction

Over the past years carbon-based nanostructured materials such as carbon, CNTs (carbon nanotubes), graphene, CNFs (carbon nanofibers), fullerenes, etc. have accomplished swift evolution and extensive use because of their Van der Waals force and covalent bonding, chemical stability, and high stiffness and strength together with their low weight.

The formation of graphene [1] raised new perspectives with respect to design of new class of materials with improved characteristics with a large diversity of essential functionalities, including physical, mechanical, thermal, and optical properties as well as chemical and bioperformance [2,3,4,5,6,7,8,9].

The sp^2^ hybridisation that occurs during the formation of carbon atoms is in charge of its stand-out in-plane elastic and mechanical properties, namely Young modulus (0.5–1 TPa [10]) and tensile strength (130 GPa [9]). Meanwhile, a large surface area of 2D structure of graphene gives a more matrix-second phase interaction area regardless of the interaction accompanied by transfer of electrons, phonons or mechanical stresses compared to CNTs or graphite [3,11]. As a result of these remarkable properties, graphene is very attractive candidate as reinforcement phase for composites with all kinds of matrix, whether ceramic, polymer or metal.

Recently, zirconia ceramics (ZrO_2_) have received significant attention from the scientific circles as a potential material for various structural applications, because of their high mechanical characteristics, physical and chemical wear, and thermal stability coupled with corrosion resistance and zero toxicity [12,13,14,15,16,17,18,19,20,21,22]. Enhancing in mechanical performance and effective control on electrical and thermal properties is a need in order for the possible future large-scale application of ZrO_2_.

It was stated that the presence of graphene phase can contribute to important improvements in bulk ceramic properties, such as mechanical, tribological, electrical, thermal, etc. There has been significant growth in the number of works on graphene-reinforced composites with concrete references to the various graphene synthesis techniques and processing methods of ceramic/graphene composites, including available sintering options after the isolation of graphene in 2004 [23,24,25,26,27,28]. The good bonded interfaces in matrix/graphene composites along with outstanding mechanical and the amazing structural features of graphene has been considered as a strengthening mechanism of graphene reinforcement.

Lately, graphene/ZrO_2_ composites were prepared using various methods. Achieving the homogeneous distribution of graphene in the initial powder is essential to produce in order to have a uniform composite upon sintering. 

For example, Liu et al. [29] and Walker et al. [30] showed that the nanostructured matrix, the GNPs (graphene nanoplatelets) generate three-dimensional cracks deflection, and the resulting in an improvement in fracture toughness. Shin and Hong fabricated reduced graphene oxide (rGO)-reinforced yttria-stabilized zirconia by spark plasma sintering, and reported a 34% increase in fracture toughness [31]. Kwon et al. demonstrated that the enhanced mechanical properties of ZrO_2_ composites can be achieved by addition of 3 wt.% graphene as a strengthening phase [32]. Chen et al. prepared graphene-reinforced zirconia ceramics using field-assisted sintering and fracture toughness values were higher in comparison to GNPs-free 3Y-TZP samples [33].

Rincón et al. produced multilaminates graphene oxide (GO)/ 8 mol% yttria stabilized zirconia by colloidal processing subsequent spark plasma sintering and reported that the addition of GO-enriched layer into zirconia laminates is slowing down the probabilities of crack generation and propagation [34].

In the work of Rao et al., the surface modified graphene zirconium oxide was synthesized and characterized as a nanocomposite material. It was utilized as an adsorbent for the removal of chlorophenols from aqueous solution. The removal efficiency was maximum towards 4-chlorophenol at pH 1.0 and decreased with increasing pH [35].

Teymourian et al. reported the synthesis of the high performance zirconium dioxide-reduced graphene oxide composite and its application as a novel architecture for electrochemical sensing and biosensing purposes [36]. 

Furthermore, Li et al. prepared zirconia/graphene nanosheets composite coatings using a plasma spraying technique. The obtained composite coating demonstrated low friction coefficient and improved wear resistance [37].

Additionally, in recent years the production of nanostructured crystalline particles has gained attention, as they possess enhanced performance abilities over conventional coarser grain materials. In order to prepare nanocrystalline zirconia-based nanocomposites, different methods such as the sol/gel method [38], vapor phase method [39], pyrolysis [40], spray pyrolysis [41], hydrolysis [42], and microwave plasma [43] have been proposed. However, these methods have many limitation factors such as complex arrangements, high temperature for the process, extended reaction time, poisonous constituents and by-products, and high costs of production, which made it difficult to prepare zirconia nanoparticles on a large-scale production. 

Therefore, in order to produce effectively isolated nanoparticles with a narrower size distribution, the hydrothermal methods are mentioned as having great potential [44,45]. This technique is straightforward and low-cost. 

All that is required for synthesis is to take a Teflon-lined stainless-steel autoclave and put the materials and precurcors in inside of it.

Uniform nucleation processes and very low grain size highlighted are highlighted as the main benefits of this method. Therefore, the purpose of this work was to produce graphene oxide (GO) with the subsequent production of reduced graphene oxide (rGO), which is used as a support to anchor zirconia nanoparticles and leading to their uniform distribution on the surface of rGO. This special nano-hybrid structure provides the opportunity to obtain a uniform composite ceramic/graphene nanopowder that can be used as a raw material to fabricate dense multi-purpose zirconia-based composites reinforced with nanostructure graphene, which combine the desired properties of each component. Thus, the aim of the present work is to obtain a nanocomposite material when there is phase uniformity and good interfacial bonding between the graphene and matrix after sintering.

## 2. Materials and Methods

### 2.1. Preparation of Graphene Oxide

Hummers’ method was used as synthesis technique of graphene oxide (GO) from graphite powder which was oxidized. Briefly, this approach involves Hummer’s reagents with low levels of NaNO_3_ and KMnO_4_ in concentrated H_2_SO_4_. Pure graphite powder was steadily added (along with NaNO_3_) into a hot concentration of H_2_SO_4_ solution that would be cooled in an ice bath. Afterward, KMnO_4_ was slowly added, in small doses, to keep the reaction temperature below 20 °C. Then the suspension was treated with a hydrogen peroxide (H_2_O_2_) solution and washed with HCl and H_2_O in order to complete the reaction with KMnO_4._ After filtration and drying, GO sheets were obtained. In the previous works, this process is decribed in further detail [28,46,47].

### 2.2. Synthesis of ZrO_2_/rGO Nanocomposite Powders

The as-prepared GO was exfoliated ultrasonically for 2 h in order to obtain a stable GO colloidal suspension with a concentration of 2.33 mg∙mL^−1^. The synthesis of reduced graphene oxide-nanozirconia (ZrO_2_/rGO) composites was carried out by means of a simple hydrothermal process, in which the GO suspension was used as the rGO precursor, N_2_H_4_·H_2_O and ZrOCl_2_·8H_2_O acted as the reducing agent and the nZrO_2_ precursor, respectively. Firstly, 40 mL of GO colloidal suspension was mixed with 20 mL of a ZrOCl_2_ solution (0.01 M) to obtain a mixed solution of GO and ZrOCl_2_ with a volume ratio of 2:1. After being sonicated for 30 min, 1 mL hydrazine hydrate was added to the mixture and was poured into a 200 mL stainless steel teflon-lined autoclave, which was sealed and maintained at 180 °C for 18 h, and then naturally cooled at room temperature. For the purpose of removing Cl-ions, the obtained material was washed, centrifuged, and re-dispersed five times in deionized water. Finally, the ZrO_2_/rGO nanocomposite powders were dried in a FreeZone2.5 freeze-drying system (LabConco, Kansas, MO, USA). The collector temperature is continuously set at −50 ± 2 °C.

Furthermore, the shell temperature and the chamber pressure were kept at 23 ± 2 °C and 0.02 ± 0.01 mbar, respectively, during the entire process [30]. For comparison purposes, non-reduced graphene oxide-nanozirconia (ZrO_2_/GO) composite powder was prepared following the same cycle without the addition of N_2_H_4_·H_2_O.

### 2.3. Microstructural Characterization of the As-Prepared Nanocomposite Powders

The microstructure, crystalline structure, and surface morphology characterizations of the as-synthesized samples were evaluated by transmission electron microscopy JEM 3010 (JEOL, Tokyo, Japan, accelerating voltage 200 kV). To this end, nanopowders were placed on a TEM grid (perforated carbon film on the copper mesh, Plano GmbH, Wetzlar, Germany) [22].

As was described previously, the particle diameter distribution of the nanocrystalline zirconia was determined from TEM micrographs by measuring the diameters of about 800 zirconia nanoparticles in both synthesized nanopowders. From these data, the density distribution of the particle diameters on number basis q_0_ and on surface area basis q_2_ were compiled. A Gaussian normal distribution was fitted to the measured distribution in order to obtain the corresponding geometric mean particle diameters μ_g_(q_0_) and μ_g_(q_2_). The cumulative distribution of the particle diameters q_0_ was fitted with a sigmoid function to obtain the characteristic particle diameter d_50_. The specific surface area S_TEM_ of the zirconia nanoparticles was calculated from μ_g_(q_2_) using the density of 5.68 g/cm^2^ and assuming spherical particles [22]. Further, the field emission scanning electron microscopy (FE-SEM) observations were performed by LYRA3 (Tescan, Brno, Czech Republic).

### 2.4. X-ray Diffraction (XRD), Raman, X-ray Photoelectron Spectroscopy (XPS) and Fourier Transform Infrared (FTIR) Characterization

X-ray diffraction measurements were conducted using an Empyrean diffractometer (PANalytical, Almelo, Netherlands) ranging from 5° to 70°. The step size was 0.05 with a scan speed of 0.06 /min. The diffractometer used Cu Kα radiation (λ = 1.5405981) working at 40 kV and with an intensity of 30 mA [48].

Raman spectra of studied materials were collected to identify the phase composition. The Raman setup is composed of a laser (DXR^TM^2 Raman Microscope, Thermo FisherScientific, Waltham, MA, USA) with a wavelength of 532 nm and a laser power of 2.0 mW. The laser beam was focused through an optical microscope’s 50× objective lens to a spot size of 2 µm on the studied area (from different spots, at an interval of 10 µm). The accumulation time for each Raman spectrum was about 10 s [26].

All X-ray photoelectron spectroscopy (XPS) measurements were carried out on a K-Alpha (Thermo FisherScientific, Waltham, MA, USA) photoelectron spectrometer equipped with a micro-focused monochromator Al Kα X-ray source. Peak fitting of the C1s and Zr3d spectra for ZrO_2_/rGO and ZrO_2_/GO nanopowders were conducted separately using a Gaussian–Lorentzian function after performing a Shirley background correction.

Fourier transform infrared (FTIR) spectra of the synthesized nanocomposite powders were measured (Vertex 70 spectrometer, Bruker AXS Inc., Madison, WI, USA) in the wavenumber range 500 cm^−1^ to 4000 cm^−1^ (transmission mode, resolution 2 cm^−1^, 120 scans per sample). For this purpose, potassium bromide (KBr) pellets (diameter 1.0–1.3 cm) of each powder sample were prepared using a uniaxial press [49].

## 3. Results and Discussion

The nanozirconia crystals have a particularly spherical shape and they are uniformly dispersed. Almost no difference was found in particle size distribution and specific surface area. The geometric mean diameters μ_g_(q_0_) and μ_g_(q_2_) of the particle size distribution are 5.1 nm ± 0.8nm and 4.8 nm ± 1.2 nm, respectively, and the d_50_ diameter is 5.0 nm with an uncertainty of ± 0.8 nm for both ZrO_2_/GO and for ZrO_2_/rGO nanopowders. The specific surface area S_TEM_ of the nanopowders was calculated from the geometric mean diameter μ_g_(q_2_) and reached a value of 110.0 m^2^/g.

**Figure 1:**
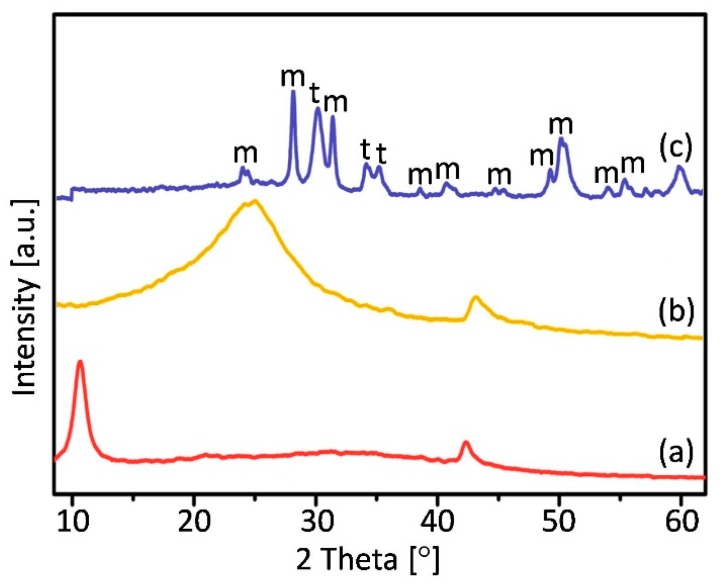
XRD patterns of (**a**) GO (**b**) rGO and (**c**) ZrO_2_/rGO materials, where “m” and “t” denote monoclinic and tetragonal zirconia, respectively.

Figure 1 shows a representative diffraction pattern of a fabricated nanopowders.

The most intense apparent peak located at 2θ = 10.8° in the curve of GO, and corresponds to the (001) reflection of GO [50]. The result confirms the oxidation and the fact that raw graphite was converted into graphene oxide becouse of the appearance of oxygen-containing functional groups [51]. The rGO pattern accompanied with a major (002) peak centered at around 25° assigned to the reduction of GO sheets and restacking into an ordered crystalline structure [52]. On the X-Ray diffractogram of ZrO_2_/rGO nanocomposite powder diffraction peaks in the region up to 20° are not presented as indicated in the case of GO and enables to say that the GO was well reduced during the reaction. In addition, analys of intensities and peaks location appearing in XRD data were compared with the infromation from the International Centre for Diffraction Data (ICDD) for tetragonal (PDF file no.01-083-0113) and monoclinic (PDF file no. 00-024-1165) structures.

**Figure 2:**
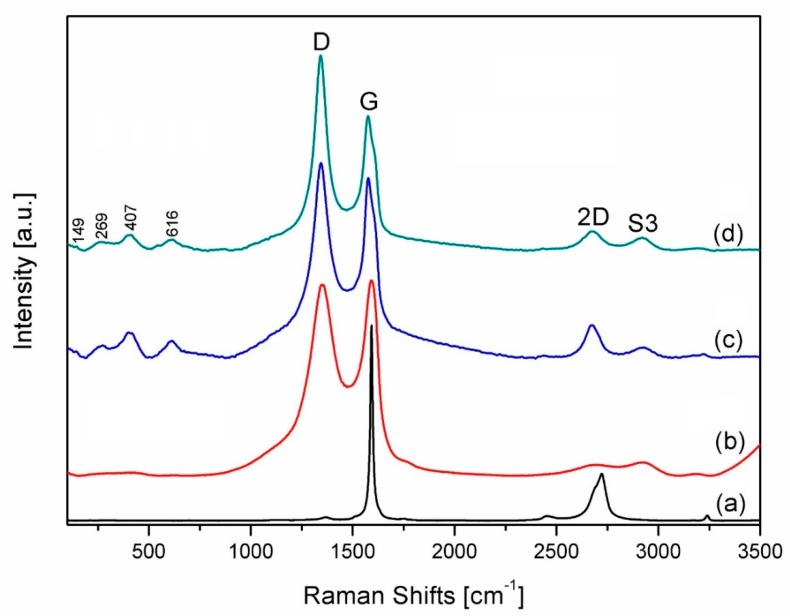
Raman spectra of starting graphite powder (**a**), graphene oxide (**b**), synthesized ZrO_2_/GO (**c**) and ZrO_2_/rGO (**d**) nanopowders. “D”, “G”, “2D” and “S3” correspond to the graphene-based structure.

The Raman spectra of all the studied materials are shown in Figure 2.

Raman spectrum of the graphite sample shows the characteristic peaks for this material. For instance, the peak located at ~1584 cm^−1^ is the G band and it is the more intence than the 2D (2720 cm^−1^) and D (~1350 cm^−1^) bands (Figure 1a). In the case of graphene (Figure 1b–d), a imperceptible second-order zone and a broad G peak are peculiarity of hybridized carbon-carbon bonds (sp^1^, sp^2^ and sp^3^) [24]. The more intense D bands (Figure 2c,d) at ~1350 cm^−1^ means that sp^2^ bonds were broken and, consequently, it indicates that a intended transformation of sp^2^ hybridization to sp^3^ was occurred, which shows a rise of the disorder in the sp^2^-hybridized carbon system [26]. This difference in intensity between D and G bands is particularly marked in ZrO_2_/rGO (Figure 2d) powders, which illustrates the creation of new defects in the sp^2^ carbon lattice during the reduction process.

It was suggested that, besides some defects in reduced graphene oxide, the presence of ZrO_2_ nanoparticles onto the surface of the rGO causes additional changes in the characteristics of the vibrations in the material’s lattice. Therefore, the intensity of D bands after the reduction process is higher in comparison to the unreduced ZrO_2_/GO nanocomposite powders. 

It should be noted that after the graphite’s chemical oxidation process, the presence of peaks attributed to 2D band were not observed in GO (Figure 2b). It can be assumed that diverse kind of oxygen-containing functional groups were embedded among the graphitic sheets in their basal plane or edges, and the presence of these groups causes the breaking of the sp^2^ carbon lattice and consequently its structural changes. However, the elimination of oxygen containing groups leads to the restoration of ordered graphitic stacking in rGO and causes the appearance of 2D peak [53,54,55].

Another band, known as conjunction between D and G intensities and appears near 2900 cm^−1^ is a second-order peak and denoted by S3.

S3/2D intesity ratio is related to the diminution in defects and involves a lower quantity of oxygen in graphene [56]. Raman shifts at 149 cm^−1^, 269 cm^−1^, 407 cm^−1^ and 616 cm^−1^ were interpreted as tetragonal zirconia phases [57,58].

**Figure 3:**
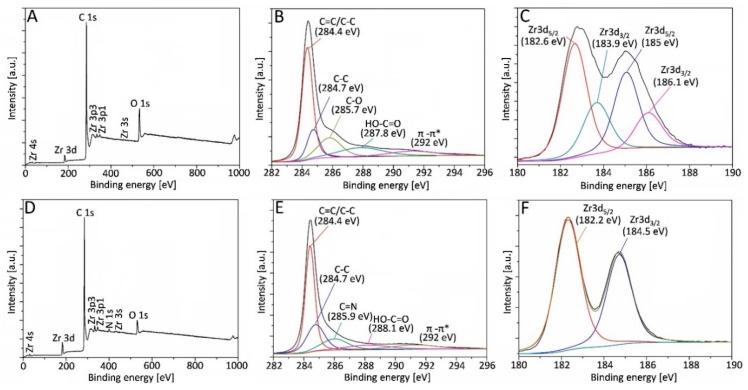
Survey XPS spectra (**A**,**D**), fitted spectra of C1s (**B**,**E**) and Zr3d (**C**,**F**) detailed scans for ZrO_2_/GO (upper row) and ZrO_2_/rGO (bottom row) powders.

To investigate the chemical changes between reduced and not reduced graphene oxide powder XPS measurements have been carried out (Figure 3).

The appearance of oxygen, zirconia, and carbon structures in the sample was confirmed by Zr3d, O1s, and C1s characteristic peaks in the full-scan XPS spectrum of ZrO_2_/GO (Figure 3A). However, the presence of an additional peak at 399.5 eV that corresponds to the nitrogen bond was detected in the hydrazine-treated ZrO_2_/rGO sample (Figure 3D). The presence of other peaks, different to Zr, oxygen, carbon and, in the case of ZrO_2_/rGO powder-nitrogen, was not found. Therefore, the processing method would be considered appropriate.

For further interpretation and to better understand the mechanism of formation and the chemical states of elements, comprehensive analysis of C and Zr XPS spectra were carried out. The detailed C1s spectra of not-reduced and chemically reduced powders are shown in Figure 3B,E, respectively. Although these obtained spectra are practically identical and show the appearance of diffent functional groups, the peak intensities in the reduced powder are smaller and consequently confirm de-oxygenation by the reduction process. The peak-fitting spectra at the binding energies of 284.4 eV, 284.7 eV and ~288 eV were assigned to carbon atoms in different functional groups: C=C/C-C (sp^2^ C) peak, C-C (sp^3^ C) peak and C=O (the carbonyl groups), respectively [59,60]. Moreover, shake-up satellites (π-π*) with a binding energy of 292 eV were observed [61,62,63], which is a characteristic satellite peak for carbon structures or aromatic compounds [64].

The occurence of these peaks confirmed the hexagonal structure of the graphene [65,66,67,68]. Meanwhile, the high resolution of the C1s spectrum of ZrO_2_/rGO powder had an additional peak at 285.9 eV (Figure 3E) may be assigned to the C in the C=N bond formation due to the presence of hydrazine [69], that is also proven by XPS survey spectrum (Figure 3D). High-resolution XPS spectral analysis for the Zr3d shows the spin-orbit splitting the components, Zr3d_3/2_ and Zr3d_5/2_, in the ZrO_2_/GO and ZrO_2_/rGO materials (Figure 3C,F, respectively). Two different chemical states of zirconia were found in the ZrO_2_/GO sample (Figure 3C). The first of them at 182.6 eV can be assigned to the zirconia sub-oxide. Meanwhile, the second one relates to the binding energy and could be determined as stoichiometric ZrO_2_ and implies that valence of zirconium remained Zr^4+^ [70,71]. The intensity of binding energy peak at 183.9 eV corresponds to the formation of the zirconia. A chi-square approximation by using a doublet with spacing between lines of 2.4 eV was obtained for ZrO_2_/rGO (Figure 3F). The position of the lines is attributed to the zirconia sub-oxide. The width of the lines (1.4 eV) demonstrates the high uniformity of the stoichiometric state of the sample. It can be assumed, comparing the spectra of both samples, that the line within the binding energy interval between 182.2 eV and 182.6 eV correlates to zirconia thin films.

**Figure 4:**
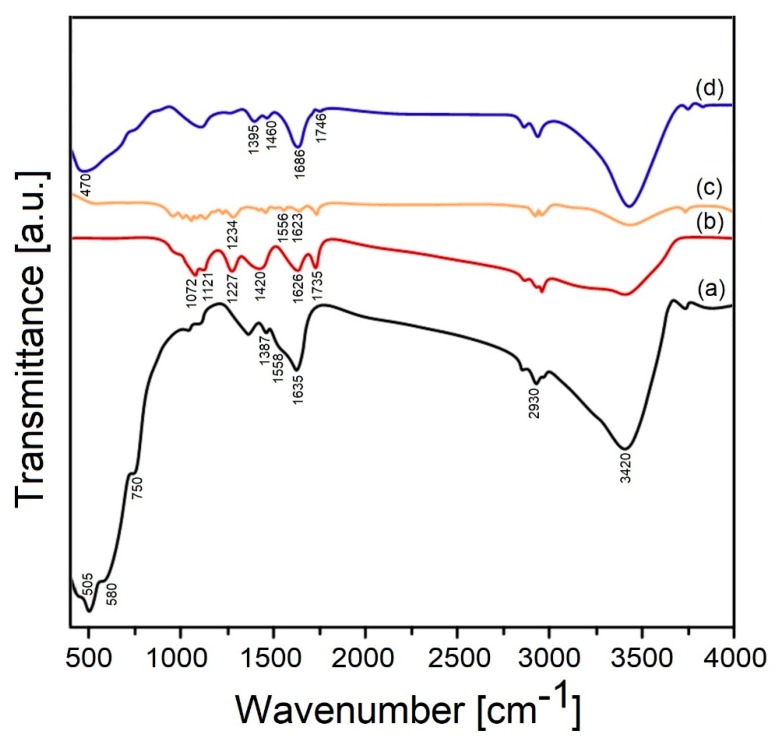
FTIR spectra of zirconia (**a**), GO (**b**), rGO (**c**) and ZrO_2_/rGO (**d**) nanopowders.

The existence of functional groups in both the raw and synthesized samples has been determined using a FTIR technique (Figure 4).

Obviously, the clear expresed absorption that appeared at peaks at 3420, 1635 and 1558 cm^−1^ correspond to the stretching (υ (−OH)) and bending (δ (−OH)) vibrations of coordinated or absorbed water molecules retained on the ZrO_2_ suface [72]. The peak appeared at 1387 cm^−1^ indicates the existence of OH deformations in the C–OH groups [60]. The Zr-O vibration of zirconia shows the intensities at 505, 580, and 750 cm^−1^ [73,74,75]. Meanwhile, the CH_2_ stretching mode demonstrates the broad absorption peaks around 2930 cm^−1^ [60].

The C=O stretching vibration of –COOH and C=O was observed at 1735 cm^−1^, while the O–H bending vibration of water molecules and the C=C skeleton stretching mode was assigned at 1626 cm^-1^ [76]. The O–H bending mode of –COOH and C–OH bonds show the intense peak at 1420 cm^−1^. The peak located at 1227 cm^−1^ belongs to the C–O–C stretching vibration. The absorption bands at 1121 and 1072 cm^−1^ corespond to the epoxy C-O and the alkoxy C-O stretching vibration, respectively [77,78,79,80,81,82,83]. Unlike the GO bands, the spectrum of rGO exhibits that the vibrational frequencies of peaks weakened and slightly vanished. The appearance of the absorption bands at 1623 and 1556 cm^−1^ was recognized to stretching of the aromatic C=C mode, while the band at 1234 cm^−1^ corresponds to the epoxy C−O and C=O, indicates that GO was reduced incompletely [72,84,85,86]. On the other hand, the characteristic band located at 1727 cm^−1^ was assigend to C=O stretching frequency belonging to the ZrO_2_/rGO nanocomposite was shifted to 1746 cm^-1^ because the relationship between the C=O group and Zr and its relative intensity was also decreased [87]. The hydrolysis of the ZrOCl_2_ solution provides the interaction between the oxygen-containing groups of GO and Zr(IV) and the appearance of two vibrational bands located at 1460 and 1395 cm^−1^, which were assigned to the creation of either a monodentate or bidentate complex [78,79,80,81]. The vibrational bands at 1686 and 470 cm^−1^ can be assigned to the C=C stretching mode and Zr–O vibration, respectively [60,72]. By this means, after the characterization of the resultant hybrid material, it can be argued that the synergies between ZrO_2_ nanoparticles and rGO nanosheets occured, i.e., ceramic nanoparticles were directly attached to the surface of the rGO nanosheets.

**Figure 5:**
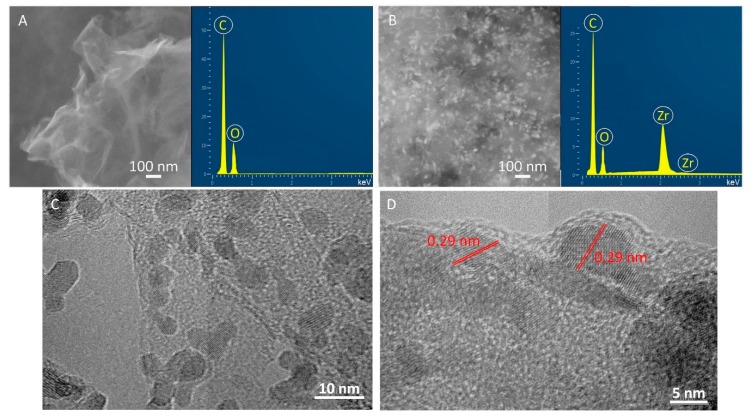
SEM images and EDS spectra of GO (**A**) and hydrothermally synthesized ZrO_2_/rGO nanocomposite powder (**B**). Representative TEM (**C**) and HRTEM (**D**) images of ZrO_2_/rGO nanocomposite powder.

Moreover, there is even further evidence to support the findings after the various characterization techniques-the SEM and TEM images where the rGO sheets and ZrO_2_ nanoparticles were seen (Figure 5). The SEM image of the GO (inset of Figure 5A) exhibited the characteristic rippled, curved and crumpled sheet-like texture, whereas the SEM image of ZrO_2_/rGO (inset of Figure 5B) showed a uniform distribution of the ZrO_2_ nanoparticles, which are anchored onto flat and not curled rGO sheets. Furthermore, the EDX spectrum of GO (Figure 5A) provided peaks of C and O, whereas the presence of C, O, and Zr in the ZrO_2_/rGO sample (Figure 5B) suggested the bonding of ZrO_2_ nanoparticles on the grapnene oxide surface. The TEM images exhibited intimate interfacial contact between rGO and ZrO_2_ nanoparticles (Figure 5C,D). The high resolution TEM image (Figure 5D) shows the lattice of 0.29 nm spacing which is very close to the lattice spacing corresponding to the (111) plane of the tetragonal structure of ZrO_2_ [36,88]. According to the above observations, it can be said that the surface GO sheets were uniformly covered by zirconia nanoparticles synthesized via hydrothermal reaction.

The development of ZrO_2_/rGO nanocomposite powders by the hydrothermal reaction technique provides the opportunity to design new materials with a large panoply of precise properties to fulfill specific requirements. In particular, for achieving the synergistic toughening effect, load-bearing capacity and high wear resistance in bulk multiphase ceramic-matrix composites for their application as engineering components [28,89]. Therefore, it is planned to focus on the fabrication of the various bulk ceramic matrix composites and to evaluate of their multifunctional properties.

## 4. Summary

In the present work, a nanostructured ZrO_2_/rGO powder was fabricated using a low-cost and simple method, namely the hydrothermal synthesis technique. This process can be summarized in a few steps: As a result of the ZrOCl_2_ solution hydrolysis process, the positively charged zirconia ions were generated and collected at a surface of negatively charged GO sheets due to electrostatic attraction. After that, the ZrO_2_/rGO nanocomposite powder was formed because of generation of nuclei, continued growth, and redox reactions in a hydrothermal process. The as-prepared nanocomposite powder was characterized by XRD, Raman, FTIR, and XPS measurements. All characterizations clearly confirmed that zirconia nanoparticles were successfully bonded into the reduced graphene oxide sheets during hydrothermal reaction. The uniformity distribution of ZrO_2_ nanoparticles, which completely cover the graphene oxide sheets, was also clearly evidenced by the SEM and TEM observations. Moreover, the lattice spacing displayed in the TEM image belongs to the tetragonal structure of zirconia with the (111) plane surface. We believe that this synthesis route will allow for the effective production of uniform ceramic/graphene nanopowders that can be used as a building block to fabricate zirconia matrix composites reinforced with a homogenous nanostructure graphene after sintering.
